# Dynamic changes in the association between maternal mRNAs and endoplasmic reticulum during ascidian early embryogenesis

**DOI:** 10.1007/s00427-021-00683-y

**Published:** 2021-12-18

**Authors:** Toshiyuki Goto, Shuhei Torii, Aoi Kondo, Junji Kawakami, Haruka Yagi, Masato Suekane, Yosky Kataoka, Takahito Nishikata

**Affiliations:** 1 Frontiers of Innovative Research in Science and Technology (FIRST), Konan University, 7-1-20 Minatojima-minamimachi, Chuo-ku, Kobe, Hyogo 650-0047 Japan; 2 Japan Testing Laboratories, Inc, Kobe, Hyogo 654-0161 Japan; 3 Laboratory for Cellular Function Imaging, RIKEN Center for Biosystems Dynamics Research, Kobe, Hyogo 650-0047 Japan; 4 Multi-Modal Microstructure Analysis Unit, RIKEN-JEOL Collaboration Center, Kobe, Hyogo 650-0047 Japan

**Keywords:** Axis determination, ER translocation, Cytoskeleton, Maternal mRNA, Translational regulation

## Abstract

Axis formation is one of the most important events occurring at the beginning of animal development. In the ascidian egg, the antero-posterior axis is established at this time owing to a dynamic cytoplasmic movement called cytoplasmic and cortical reorganisation. During this movement, mitochondria, endoplasmic reticulum (ER), and maternal mRNAs (postplasmic/PEM RNAs) are translocated to the future posterior side. Although accumulating evidence indicates the crucial roles played by the asymmetrical localisation of these organelles and the translational regulation of postplasmic/PEM RNAs, the organisation of ER has not been described in sufficient detail to date owing to technical difficulties. In this study, we developed three different multiple staining protocols for visualising the ER in combination with mitochondria, microtubules, or mRNAs in whole-mount specimens. We defined the internally expanded “dense ER” using these protocols and described cisterna-like structures of the dense ER using focused ion beam-scanning electron microscopy. Most importantly, we described the dynamic changes in the colocalisation of postplasmic/PEM mRNAs and dense ER; for example, *macho-1* mRNA was detached and excluded from the dense ER during the second phase of ooplasmic movements. These detailed descriptions of the association between maternal mRNA and ER can provide clues for understanding the translational regulation mechanisms underlying axis determination during ascidian early embryogenesis.

**Supplementary Information:**

The online version contains supplementary material available at 10.1007/s00427-021-00683-y.

## Introduction

The morphogenesis of animal embryos begins with the formation of body axes, namely the antero-posterior and dorso-ventral axes. An asymmetrical localisation of maternal determinants (proteins and/or mRNAs) is essential for the formation of these body axes. In frog eggs, the dorso-ventral axis is established by the asymmetrical localisation of maternal determinants, including *wnt-11* mRNA and dishevelled proteins, driven by cortical rotation (Miller et al. 1999; Tao et al. 2005). During the early development of *Caenorhabditis elegans*, cytoplasmic flow leads to the asymmetrical localisation of cytoplasmic determinants such as PIE-1 protein and P-granules (Lyczak et al. 2002; Nance and Zallen 2011). Thus, the asymmetrical localisation of maternal determinants is conserved in a wide variety of species, with different species-specific determinants and localisation patterns.

In the ascidian egg, the movement of the myoplasm (containing mitochondria, endoplasmic reticulum (ER), maternal mRNAs, and characteristic colour pigment in certain species), called cytoplasmic and cortical reorganisation or the ooplasmic movement, is responsible for the antero-posterior axis determination and muscle differentiation (Conklin 1905; Nishida 1994; Sardet et al. 2003; Prodon et al. 2007). The ooplasmic movement comprises two phases: immediately after fertilisation, the cortical actin network contracts towards the vegetal pole and causes a translocation of the myoplasm and sperm nucleus to the vegetal pole (first phase; Sawada and Schatten 1989; Chiba et al. 1999; Roegiers et al. 1999). Approximately 30–50 min after fertilisation, the sperm aster migrates towards the posterior pole and then to the centre of the egg to fuse with the female nucleus (Roegiers et al. 1999; Sardet et al. 2007). This movement of the sperm aster contributes to the translocation of the myoplasm from the vegetal pole to the future posterior pole (second phase; Sawada and Schatten 1989; Chiba et al. 1999; Roegiers et al. 1999). During these movements, the cortical region of the myoplasm contains the ER, designated as cortical ER (cER) by Sardet et al. (1992), and the inner region is occupied by mitochondria-rich cytoplasm (MRC).

Approximately 40 maternal mRNAs localised in the myoplasm have been identified and designated as postplasmic/PEM RNAs (Prodon et al. 2007). These postplasmic/PEM RNAs are classified into two types, I and II, according to their localisation patterns (Sasakura et al. 2000; Prodon et al. 2007). Type I postplasmic/PEM mRNAs, such as *macho-1*, are localised to and translocate with the myoplasm (Nishida 2005). In contrast, type II mRNAs, such as *vasa*, are evenly distributed in the unfertilised egg cytoplasm but localise to the posterior pole region of the myoplasm after the second phase of movement (Shirae-Kurabayashi et al. 2006; Prodon et al. 2007). This posterior localisation of postplasmic/PEM RNAs persists during the cleavage stages, with predominant localisation to the centrosome attracting body (CAB), which is rich in ER and is required for the unequal cleavages occurring from the 8- to 64-cell stages that manifest the antero-posterior asymmetry (Nishikata et al. 1999; Sardet et al. 2003).

Type I postplasmic/PEM RNAs are colocalised with the cER, ribosomes, and several regulators that are required for translational initiation during the first phase of movement and cleavage stages (Prodon et al. 2005; Paix et al. 2009, 2011). Moreover, after observing the results of hypotonic treatment on the isolated cortex, Sardet et al. (2003) strongly suggested that type I postplasmic/PEM mRNAs are anchored to the cER. These reports suggested that the translational regulation of postplasmic/PEM RNAs is conducted on the cER.

Although a previously developed live imaging method used to visualise the ER using a microinjection of carbocyanine dye clarifies its movement (Speksnijder et al. 1993; Prodon et al. 2005), its detailed organisation and associations with other organelles are not clearly visible. In contrast, isolation of the cortex is a feasible method for visualising the cER clearly, even with electron microscopy, and analysing its associations with the postplasmic/PEM RNAs (Sardet et al. 2003; Prodon et al. 2005). However, the spatial information was restricted to very thin layers (less than 1.0 µm) of the fragmented egg cortex during earlier attempts (Prodon et al. 2005). Thus, a novel method that enables the simultaneous visualisation of the entire ER and postplasmic/PEM RNAs in whole-mount specimens is desirable.

In this study, we developed three different multiple staining protocols for the visualisation of ER in combination with mitochondria, microtubules, or mRNAs, each with sufficient resolution to reveal the entire spatio-temporal (four-dimensional) pattern within the myoplasm during the ooplasmic movement, using whole-mount specimens. This enabled us to observe the relative positioning of the ER/mitochondria, ER/microtubules, ER/mRNAs, and ER/mitochondria/mRNA in detail. Moreover, we described the ultrafine ER structure within the myoplasm using focused ion beam-scanning electron microscopy (FIB-SEM) for the first time. According to our detailed observations, we designated the characteristic ER mass within the myoplasm as “dense ER,” which was considered to correspond to the cER, but expanded its localisation deeper into the subcortical region during the latter half of the first cell cycle. Furthermore, our observations revealed dynamic changes in the colocalisation of the dense ER and postplasmic/PEM RNAs during the ooplasmic movement and early cleavage stages, suggesting intricate translational regulation of maternal mRNAs and their importance in antero-posterior axis formation during ascidian early development.

## Materials and methods

### Animal experiments

Ascidian (*Ciona intestinalis* type A; also called *Ciona robusta*) adults were obtained from the National BioResource Project (NBRP), Japan. Methods for egg and sperm handling, fertilisation, and dechorionation were followed as described previously (Ishii et al. 2012, 2014). The embryos were reared in filtered seawater at 18 °C. At this temperature, the first and second movement phases occur immediately after fertilisation and approximately 30-min post-fertilisation (mpf), respectively, and the first cleavage occurs at approximately 60 mpf. Detailed staging of the first cell cycle followed a previous report (Goto et al. 2019). The process was divided into 15 stages (metaphase, anaphase, and telophase of meiotic division I [Meta I, Ana I, and Telo I]; prophase, prometaphase, metaphase, anaphase, and telophase of meiotic division II [Pro II, Prometa II, Meta II, Ana II, and Telo II]; pronuclear formation, pronuclear migration, and pronuclear fusion [PNfo, PNm, and PNfu]; prometaphase, metaphase, anaphase, and telophase of 1st mitotic division [Prometa, Meta, Ana, and Telo]) according to the shape of nucleus/chromosomes and mitotic apparatus. The unfertilised egg was defined as normalised time 0 and the stages Meta I, Telo I, PNfo, PNfu, and Meta roughly correspond to normalised times of 0.2, 0.6, 0.75, and 0.9, respectively. The end of Telo, which is the start of 2-cell stage, was defined as normalised time 1.

### Microinjection

The microinjection equipment comprised a micromanipulator (Model MN-151; Narishige, Tokyo, Japan), microscope (Stemi 2000-c; Carl Zeiss, Oberkochen, Germany), and glass capillary (Microcaps, Broomall, PA). Dechorionated unfertilised eggs were aligned on a 1.2% agar-coated dish. The eggs were then injected with 1,1′-dioctadecyl-3,3,3′,3′-tetramethylindocarbocyanine perchlorate (DiIC18(3); Thermo Fisher Scientific, Waltham, Massachusetts) saturated in soybean oil (Ajinomoto, Tokyo, Japan).

### Whole-mount immunofluorescence

For double staining the ER and mitochondria, dechorionated *Ciona* eggs/embryos were fixed with Fix solution 1 (modified from Prodon et al. 2009; 3.2% formaldehyde (Polysciences Inc., Warrington, Pennsylvania), 100 mM HEPES (pH = 7.0), 50 mM EGTA, 10 mM MgSO_4_, and 525 mM sucrose) for 1 h at room temperature (approximately 25 °C). For double staining the ER and microtubules, dechorionated *Ciona* eggs/embryos were fixed with Fix solution 2 (3.2% formaldehyde in 80% methanol) at − 30 °C for 1 h, followed by continued fixation at room temperature for 1 h with gentle shaking every 20 min. Both types of fixed specimens were treated with ethanol up series (35%, 70%, and 100%) and stored at − 30 °C until further use. After washing with phosphate-buffered saline (PBS) containing 0.05% Tween 20 (PBST), the specimens were treated with G1T0 (Ishii et al. 2017; 4 mol/L urea (MP Biomedicals, Santa Ana, California), and 1% glycerol in distilled water) for 90 min at 4 °C, and then treated with antigen retrieval solution (modified from Hayashi et al. 2011; 6 M urea and 0.1 M Tris–HCl, pH 9.5) for 30 min at 80 °C. The specimens were immunostained with the following antibodies: anti-NN18 mouse monoclonal antibody (anti-neurofilament antibody; Sigma-Aldrich, St. Louis, Missouri; 1:100 dilution), anti-α-tubulin mouse monoclonal antibody (anti-microtubule antibody; clone DM1A; Sigma-Aldrich; 1:100 dilution), anti-glucose-regulated protein 78 (GRP78; also known as Bip) rabbit polyclonal antibody (anti-ER antibody; StressMarq Biosciences, Victoria, BC, Canada; 1:100 dilution), Alexa Fluor 488-conjugated goat anti-mouse IgG antibody (Thermo Fisher Scientific; 1:1000 dilution), and Alexa Fluor Plus 555-conjugated goat anti-rabbit IgG antibody (Thermo Fisher Scientific; 1:1000 dilution). The NN18 antibody is a good marker of mitochondria to recognise the F1-ATP synthase alpha-subunit in *Ciona* (Chenevert et al. 2013). The nuclei were stained with 5 μg/ml 4′,6-diamidino-2-phenylindole (DAPI). The stained specimens were mounted with methyl salicylate (Nacalai Tesque, Kyoto, Japan).

### Conventional RNA fluorescence in situ hybridisation

For fluorescence in situ hybridisation*,* dechorionated *Ciona* eggs/embryos were fixed with 4% paraformaldehyde and 0.5 M NaCl in 0.1 M 3-(N-morpholino)propanesulfonic acid (MOPS: pH = 7.5) for 1 h at room temperature. The fixed specimens were treated with ethanol up series (35%, 70%, and 100%) and stored at − 30 °C until further use. Sense and antisense RNA probes were transcribed from *Ciona* cDNA clones (Ghost; Satou and Satoh 2005), *ci-macho-1* (cieg016n12), *ci-pem-1* (cieg001j23), or *ci-vasa* (citb056g04), using T7 and T3 RNA polymerases (Sigma-Aldrich), respectively, with DIG RNA Labeling Mix (Sigma-Aldrich). The fixed specimens were washed with PBST and treated with 5 µg/ml proteinase K for 15 min at 37 °C. The specimens were then post-fixed with 4% paraformaldehyde for 1 h at room temperature, followed by treatment with 0.1 M 2,2′,2′-nitrilotriethanol (Fujifilm-Wako, Tokyo, Japan) and 0.27% acetic anhydride (Nacalai Tesque) for 10 min at room temperature. Pre-hybridisation buffer was prepared by mixing 50% formamide, 50 µg/ml heparin, 100 µg/ml yeast tRNA, and 1% Tween 20. The specimens were hybridised with 0.5 µg/ml RNA probes in the prepared buffer for 16 h at 50 °C after 1 h of pre-hybridisation. After hybridisation, the specimens were washed with SSC (5 × , 2 × , and 0.2 ×). DIG was detected using a horseradish peroxidase-conjugated anti-DIG Fab fragment (Sigma-Aldrich; 1:100 dilution). Signals were enhanced using Cy5-Tyramide (Akoya Biosciences, Marlborough, Massachusetts).

### Whole-mount double staining with immunofluorescence and fluorescence in situ hybridisation

Dechorionated *Ciona* eggs/embryos were fixed with Fix solution 2, and rehydrated specimens were treated with 0.2% Triton X-100 in PBS for 10 min at room temperature. The specimens were post-fixed with 4% paraformaldehyde for 1 h at room temperature, followed by treatment with 0.1 M 2,2′,2′-nitrilotriethanol and 0.27% acetic anhydride for 10 min at room temperature. The specimens were treated with G1T0 followed by antigen retrieval with a modified solution (containing 3.78 M urea, 0.063 M Tris–HCl [pH 9.5], 50 µg/ml heparin, 100 µg/ml yeast tRNA, and 1% Tween 20). The specimens were hybridised with an RNA probe in pre-hybridisation buffer for 16 h at 50 °C. The specimens were then treated sequentially with freshly prepared appropriate primary and secondary antibody sets. After immunostaining, the in situ hybridisation signals were enhanced with Cy5-Tyramide.

### Double staining with immunofluorescence and fluorescence in situ hybridisation of the isolated cortex

Isolated cortex was prepared as previously described (Sardet et al. 1992, 2011). Dechorionated *Ciona* eggs/embryos along with calcium-free artificial sea water (CaF-ASW; 0.46 M NaCl, 9.4 mM KCl, 0.1 M MgSO_4_, 5.9 mM NaHCO_3_, and 0.2 mM EDTA) were placed on a poly L-lysine-coated glass coverslip. The eggs/embryos were settled for 2 min, and then CaF-ASW was carefully replaced by isotonic buffer X (350 mM K-aspartate, 130 mM taurine, 170 mM betaine, 50 mM glycine, 19 mM MgCl_2_, and HEPES (pH = 7.0)]. The attached *Ciona* eggs/embryos were gently sheared using a stream of buffer X.

For conventional staining, prepared isolated cortices were fixed with 3.7% formaldehyde and 0.1% glutaraldehyde in CIM buffer (Sardet et al. 2011; 800 mM glucose, 100 mM KCl, 2 mM MgCl_2_, 5 mM EGTA, and 10 mM MOPS (pH = 7.0)) for 30 min at room temperature. The specimens were immunostained with the following antibodies: anti-ATP5B chicken antisera (anti-ATP synthase β-subunit antisera; Sigma-Aldrich; 1:100 dilution), anti-α-tubulin mouse monoclonal antibody, horseradish peroxidase-conjugated anti-DIG Fab fragment, Alexa Fluor 405-conjugated goat anti-mouse IgG antibody (Thermo Fisher Scientific; 1:200 dilution), and Alexa Fluor 633-conjugated goat anti-chicken IgG antibody (Thermo Fisher Scientific; 1:1000 dilution). The ATP5B antisera are a good marker of mitochondria to recognise the F1-ATP synthase beta-subunit in *Ciona* (Ishii et al. 2012). After immunostaining, conventional in situ hybridisation was performed as described above, but without treatment with detergent or proteinase K. The signals were enhanced with FITC-Tyramide, and the ER was labelled with a diluted solution (1:100) of 1,1′-dioctadecyl-6,6′-di(4-sulfophenyl)-3,3,3′,3′-tetramethylindocarbocyanine (SP-DiIC18(3); Thermo Fisher Scientific) saturated in DMSO.

For staining with our method, cold Fix solution 2 (–30 °C) was applied to the isolated cortex, which was then incubated for 30 min at room temperature. The protocols for immunostaining and in situ hybridisation were the same as our method for whole-mount specimens except for the antibodies: anti-ATP5B chicken antisera and Alexa Fluor 633-conjugated goat anti-chicken IgG antibody for mitochondria staining, and anti-α-tubulin mouse monoclonal antibody and Alexa Fluor 405-conjugated goat anti-mouse IgG antibody for microtubule staining.

#### FIB-SEM

Dechorionated *Ciona* eggs/embryos were fixed with 2.5% glutaraldehyde (Nacalai Tesque) containing Fix solution 1 for 1 h at room temperature. The fixed specimens were treated with ethanol up series (35%, 70%, and 100%) and stored at − 30 °C until further use. The specimens were post-fixed in 2.0% osmium tetroxide (Nisshin-EM, Tokyo, Japan) and 1.5% potassium ferrocyanide in artificial sea water (420 mM NaCl, 9 mM KCl, 10 mM CaCl_2_, 24.5 mM MgSO_4_, 2.15 mM NaHCO_3_, HEPES [pH = 8.0]) for 1 h at 4 °C. The post-fixed specimens were then treated with 1% thiocarbohydrazide (Tokyo Chemical Industry, Tokyo, Japan) for 20 min at room temperature. The specimens were post-fixed again in 2.0% osmium tetroxide for 30 min at room temperature. The specimens were stained with 3% Ti-blue (Nisshin-EM) for 16 h at 4 °C. Next, the specimens were stained with 0.03 M aspartic acid and 0.02 M lead nitrate for 30 min at 60 °C. After dehydration with EtOH up series and acetone, the specimens were embedded in epoxy resin (CLEARPOXY Resin, Sankei Co., Ltd, Tokyo, Japan). The specimens were observed using FIB-SEM (NX5000; Hitachi, Tokyo, Japan). More than 40 eggs were observed in the present study.

### Image acquisition and data analysis

The specimens were observed under LSM700 (Carl Zeiss AG, Jena, Germany) or A1RHD25 (Nikon, Tokyo, Japan) confocal microscope using ZEN (Carl Zeiss) or NIS element imaging software (Nikon), respectively. The vertical and crosswise directions in the displayed mid-plane images corresponded to the animal-vegetal and antero-posterior axes, respectively. The animal pole was defined based on the position of the meiotic apparatus, myoplasm configuration, or parallel direction of the *c*ortical *a*rray of *m*icrotubules in the *p*osterior-vegetal region (CAMP; Ishii et al. 2017). All analyses were performed using the ImageJ Fiji software (Schindelin et al. 2012). Regions of dense ER, MRC, and CAMP were extracted using enhanced contrast and binarisation, and their centres of mass were calculated. The angles between these centres of mass and the animal-vegetal axis were measured using a single optical section of the mid-plane. To analyse the colocalisation between the ER and mRNA signals, 3D models of the mid-plane or CAB-forming region were first rendered from five optical sections. Then, the dense ER region was extracted using denoising, contrast adjustment, and binarisation, followed by a restriction of the object size. The maternal mRNA-positive region was extracted using binarisation of the mRNA signals. The ratio of the mRNA-positive regions on the dense ER to the total area of mRNA-positive regions was calculated. The parameters for the extraction of each dense ER or mRNA were fixed for all specimens. All the experiments were repeated independently at least twice, mostly more than five times. For quantitative analyses, only the specimen that was exactly staged and included both the animal-vegetal/antero-posterior axes or both the antero-posterior/left–right axes in a single optical section was used.

## Results

### Emergence of dense ER in the myoplasm and translocation to the posterior side

**Fig. 1:**
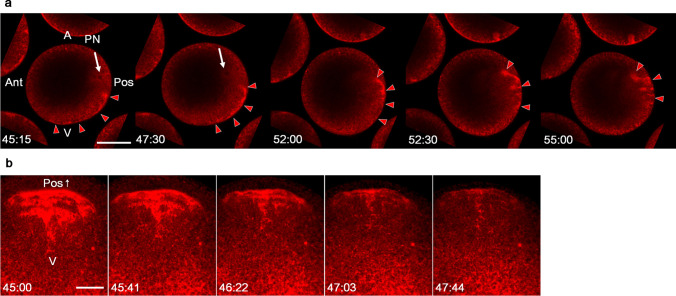
The structure and translocation of ER during the second phase of movement. **a** Mid-plane optical sections of DiIC18 (red)-injected living eggs from 45 to 55 mpf (mm:ss). This period corresponds to the period from PNfu to Meta. White arrows and red arrowheads indicate the pronucleus (PN) and dense ER, respectively. Animal pole (A) is up. Anterior pole (Ant) is left; posterior pole (Pos) is right. **b** The 3D model of approximately two-third of vegetal hemisphere was rendered from optical sections of DiIC18 (red)-injected living eggs at approximately the PNfu stage (from 45:00 to 47:44 after fertilisation). The posterior half of the vegetal views is represented here. The dense ER moved posteriorly. V, vegetal pole; Pos↑, posterior side. Six independent experiments were carried out. In each experiment, appropriately oriented egg was selected from more than 50 DiIC18-injected living eggs. Scale bar: 50 µm

To re-examine the translocation of ER during the second phase of movement, fertilised eggs were injected with carbocyanine dye (DiIC18(3)) and observed using time-lapse confocal imaging. Although the background staining of the ER covered the entire egg cytoplasm, a densely stained ER mass was clearly identified within the myoplasm. This ER mass was translocated to the posterior pole along the vegetal cortex and several portions, including its leading tip, intruded into the deeper cytoplasm around the posterior pole (Fig. 1a and Online Resource 1, Mov. S1). According to the posterior views of the intruding ER (Fig. 1b and Online Resource 2, Mov. S2), it appeared to be a parallel fold of flattened ER mass and relatively continuous beneath the cortical region of the mid-plane. These observations are consistent with those in previous studies (Speksnijder et al. 1993; Prodon et al. 2005). Although the ER network beneath the egg cortex, namely the cER (Sardet et al. 1992), has been well studied to date, such a deeply intruded ER mass has not been properly characterised. Thus, to reveal the likely function of ER at the beginning of ascidian development, we focused on the characterisation of this ER mass, designated as “dense ER” in this study.

For a detailed characterisation of the dense ER, multiple staining methods for fixed whole-mount specimens are required. We developed an immunostaining method to visualise the dense ER within whole-mount specimens with sufficient resolution using polyclonal antibodies against Bip, a molecular chaperone that is abundantly present in rough ER (Bole et al. 1989; Terasaki and Reese 1992; Ibrahim et al. 2019). We used the immunostaining protocols in conjunction with a modification of the urea-based antigen retrieval protocol (Hayashi et al. 2011). Moreover, we optimised this method for double-, triple-, or quadruple-staining with other organelles to obtain direct information about their colocalisation.

**Fig. 2:**
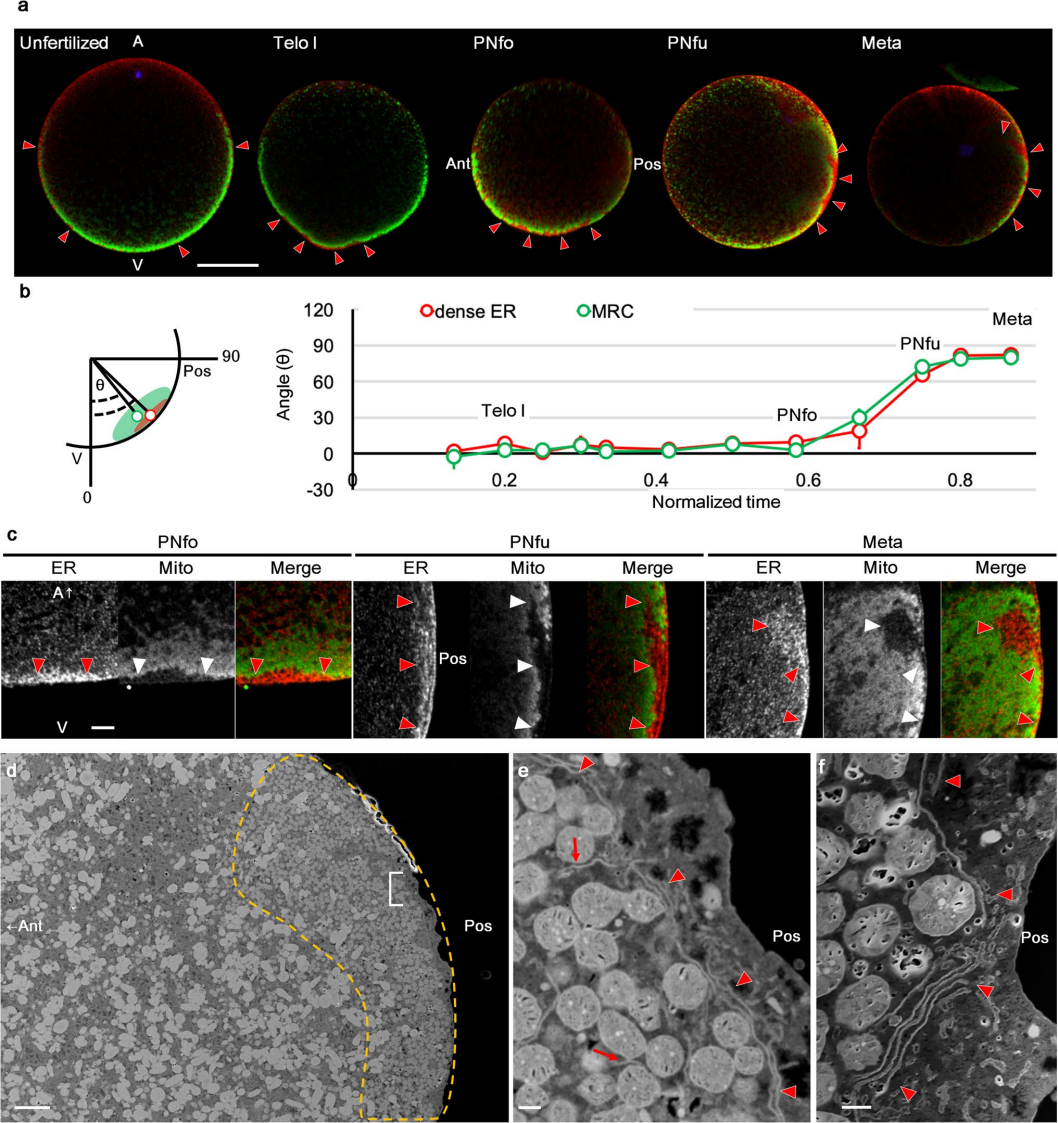
Discrete distribution of ER and mitochondria during the first cell cycle. **a** Our triple-staining protocol for nucleus (blue), ER (red), and mitochondria (green) was applied to the unfertilised eggs (unfertilised) and eggs of Telo, PNfo, PNfu, and Meta. A single optical section of the mid-plane is shown. Animal pole (A) is up. The antero (Ant)-posterior (Pos) axis becomes evident from PNfo stage owing to the position of the sperm aster. Red arrowheads indicate dense ER regions. Scale bar: 50 µm. **b** The movement of dense ER (red) and mitochondria-rich cytoplasm (MRC; green) from the vegetal pole (V) to the posterior pole (Pos) were quantitatively measured, as shown in the schema. The angles (θ) between the centre of the dense ER or MRC (red or green circles, respectively) and animal-vegetal axis were measured and represented as a line graph. Error bars represent SD (*n* = 3). **c** High-magnification images of dense ER regions at PNfo, PNfu, and Meta stages (as indicated on the top). The ER, mitochondria, and merged fluorescence channels are separately shown (as indicated on the top). Dense ER regions correspond to mitochondria-free regions (white arrowheads). Notably, the dense ER at PNfu displays a striped pattern. Approximately ten confocal images were acquired in each stage of cell cycle progression from five independent experiments. Scale bar: 5 µm. **d** Focused ion beam-scanning electron microscope (FIB-SEM) image of posterior pole region at the PNfu stage with myoplasmic region (yellow broken line), comprising MRC and dense ER regions (red arrowheads). Scale bar: 6 µm. **e**, **f** Enlarged images of dense ER (E; indicated by “[” in d, f; another specimen) show the cisterna-like structure, which is a stacked ER sheet (red arrowheads). Dense ER is almost mitochondria-free. Loosely extended ER (red arrow) can be observed in the MRC region. More than 40 eggs were observed in wide-field view, then appropriately oriented three FIB-SEM images were acquired. Scale bars: 0.6 µm

First, we described the movement of the dense ER and MRC along with the meiotic or mitotic cycles, as shown in Fig. 2a–c and Online Resource 3, Fig. S1. The cell cycle progression corresponded precisely with changes in the ooplasmic movement, as described by Goto et al. (2019). The ER staining in the unfertilised egg cortex was thin, and condensed ER was clearly observed at the vegetal pole after the first movement. These staining patterns strongly suggested that the dense ER was almost identical to the cER, as suggested by Sardet et al. (1992). During the second phase of movement, the dense ER moved to the posterior pole in coordination with the movement of the MRC (Fig. 2a, b; PNfo to Meta). These movements were similar to that of cER, although the dense ER intruded deeper (5–10 µm) from the PNfo stage (approximately 35 mpf) onwards. According to the high-magnification images (Fig. 2c), weak dot-like staining of the ER was observed in the MRC, suggesting that a considerable amount of ER continued from the dense ER, designated as loosely extended ER in this study. On the other hand, the boundary of the MRC was evident during the entire ooplasmic movement. This indicated that mitochondria were rare in the dense ER region, which may be one of the characteristics that distinguish dense ER from loosely extended ER.

The ultrastructure of the dense ER during the second phase of movement was observed using FIB-SEM. The widefield mid-plane image clearly shows the myoplasm composed of mitochondria and ER (dotted line: Fig. 2d). High-magnification images showed that the dense ER at this stage mainly comprised a cisterna-like ER structure and ER sheets stacked in a parallel array (Fig. 2e, f). This cisterna-like ER structure appeared to correspond to the stripe-shaped staining of the dense ER at the PNfu stage (Fig. 2c), suggesting a transiently formed structure.

### Associated movement of dense ER with the cortical microtubule structure during second phase of movement

**Fig. 3:**
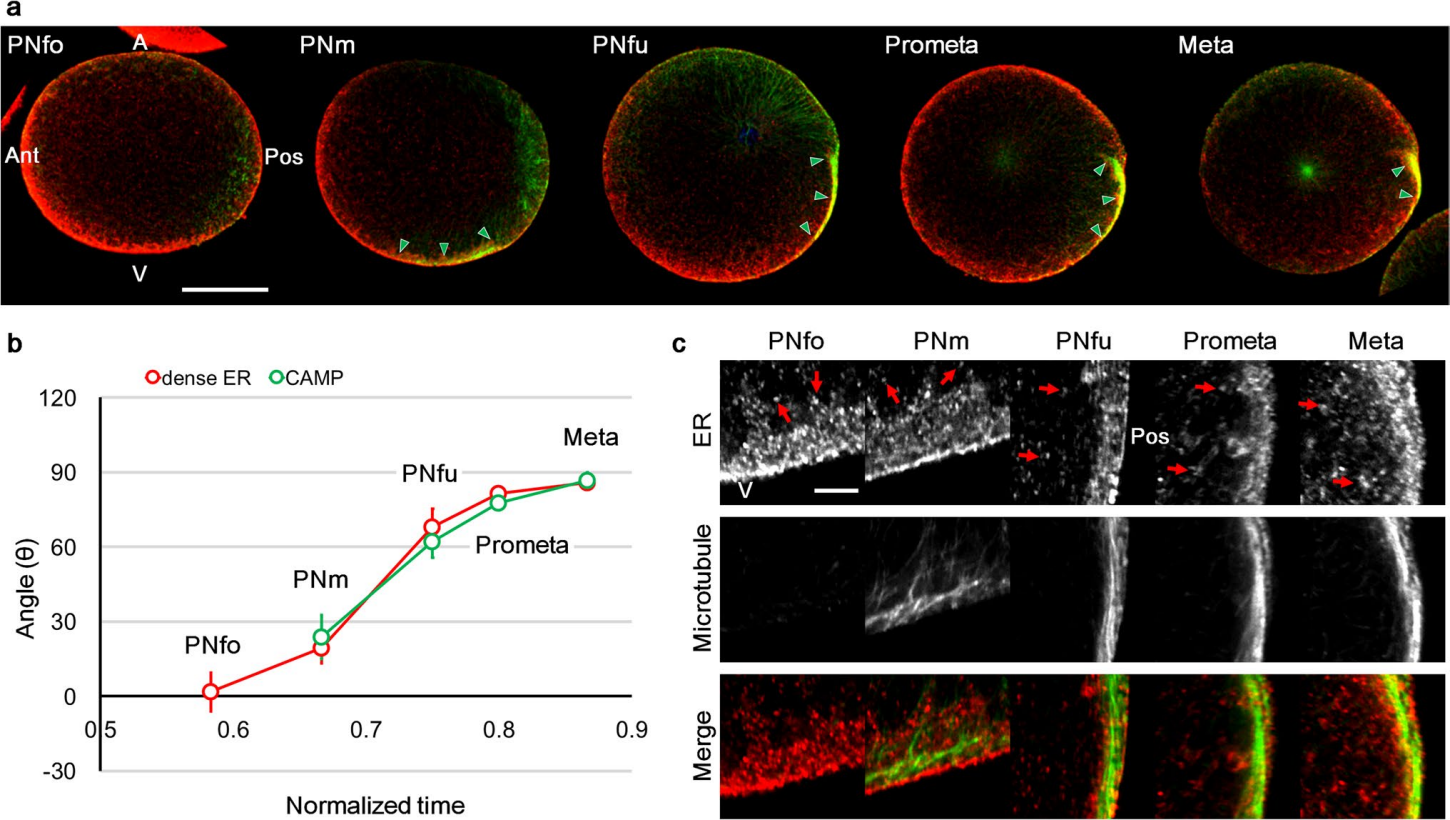
Colocalisation of ER and cortical array of microtubules in posterior-vegetal region (CAMP) during second phase of movement. **a** Another novel triple-staining protocol for nucleus (blue), ER (red), and microtubules (MT: green) was applied to the eggs during PNfo, PNm, PNfu, Prometa, and Meta. A single optical section of the mid-plane is shown. In this method, although the border of dens ER and loosely extended ER was not obvious in the lower magnification images because of the relatively bright staining of entire ER, it was identifiable in the high-magnification image of thin optical section. CAMP (green arrowheads) was observed from PNm stage within the dense ER region. Scale bar: 50 µm. **b** The movement of dense ER and CAMP was quantitatively analysed as described in Fig. 2. The angles (θ) between the centre of the dense ER or CAMP (red or green circles, respectively) and animal-vegetal axis were measured and represented as a line graph. Error bars represent SD (*n* = 3). **c** High-magnification images of CAMP at PNfo, PNm, PNfu, Prometa, and Meta (as shown on the top). ER, microtubules, and merged fluorescence channels are indicated on the left side. Approximately 30 confocal images were acquired in each stage of cell cycle progression from five independent experiments. Scale bar: 5 µm. Animal pole (A) is up and posterior pole (Pos) is at the right in all photographs. Anterior pole (Ant) and vegetal pole (V) are indicated in a few photos

We previously reported a cortical microtubule structure, CAMP (Ishii et al. 2017), which was a parallel array of microtubules in the posterior-vegetal region observed during the second phase and suggested to have a major contribution to the myoplasmic movement. We performed double immunostaining for ER and microtubules in whole-mount specimens to observe their colocalisation. Our newly invented cold alcohol-formaldehyde fixation (− 30 °C) enabled the observation of both the dense ER and microtubule structures (Fig. 3 and Online Resource 3, Fig. S2). The CAMP was formed within the dense ER region, and these two moved together during the second phase of movement (Fig. 3a, b: PNm to Meta and Online Resource 3, Fig. S2). Although the relatively bright staining of loosely extended ER concealed the border of dense ER in lower magnification images because of its thick optical section, higher magnification and thin optical images revealed the dense ER (Fig. 3c). Microtubule bundles were formed within the intricate ER network, along with the transiently formed striped pattern of the ER at the PNfu stage (Fig. 3c). These results suggest that the CAMP contributes to the posterior translocation of dense ER.

### Temporal dissociation of postplasmic/PEM RNAs and dense ER during second phase of movement

**Fig. 4:**
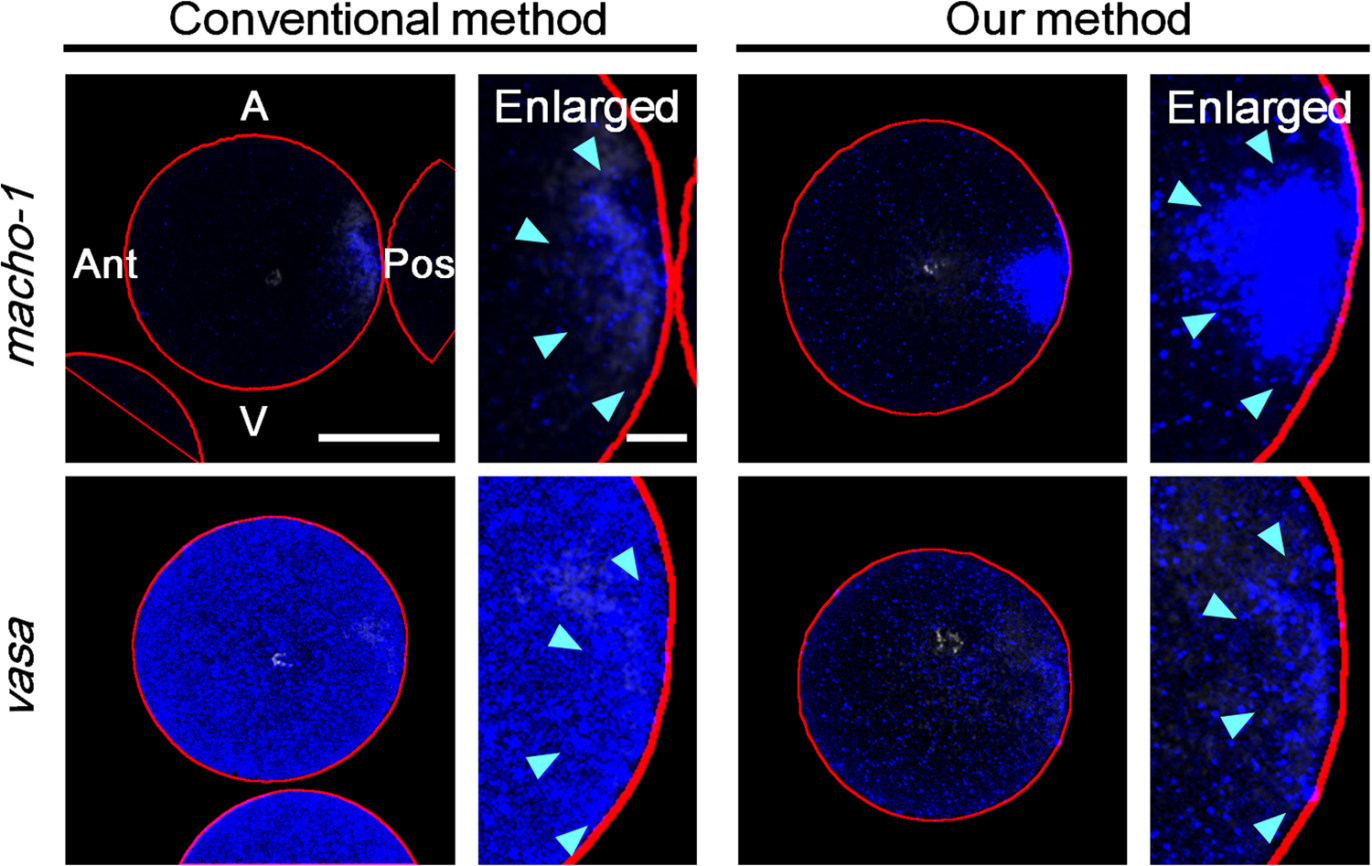
Comparison of two fluorescence in situ hybridisation protocols. Conventional and our methods of in situ hybridisation were compared with two different probes, *macho-1* and *vasa*, under the same conditions: tyramide-fluorescent detection system and confocal microscopy with same image acquisition settings. Posteriorly localised signals were detected with both probes (blue: light blue arrowheads). These eggs were fixed in metaphase in first mitosis. Optical sections of the mid-plane are shown. Animal pole (A) is up and posterior pole (Pos) is at the right in all photographs. Anterior pole (Ant) and vegetal pole (V) are also indicated. The outline of the egg detected by ImageJ is indicated by a red line. Compared to the conventional method, our method achieved high signal intensity with the *macho-1* probe and a marked reduction of the cytoplasmic background with the *vasa* probe. Approximately ten confocal images were acquired from three independent experiments. Scale bars: 50 µm (entire egg image) and 10 µm (enlarged image)

To reveal the mRNA and dense ER colocalisation, we developed a new method that combines fluorescence in situ hybridisation and immunostaining for ER, mitochondria, and microtubules. First, we performed a validation study to compare the conventional and our novel fluorescence in situ hybridisation methods under the same image acquisition conditions (Fig. 4). Using *macho-1* as a probe for type I postplasmic/PEM RNA, our method reproduced the posterior localisation pattern, also observed previously using the conventional method, but with stronger signal intensity. Using *vasa* as a probe for type II postplasmic/PEM RNA, our method revealed posterior localisation patterns in higher contrast. Thus, we concluded that our method enabled the observation of clear and precise signals of *macho-1* and *vasa* and recapitulated the prior observations.

**Fig. 5:**
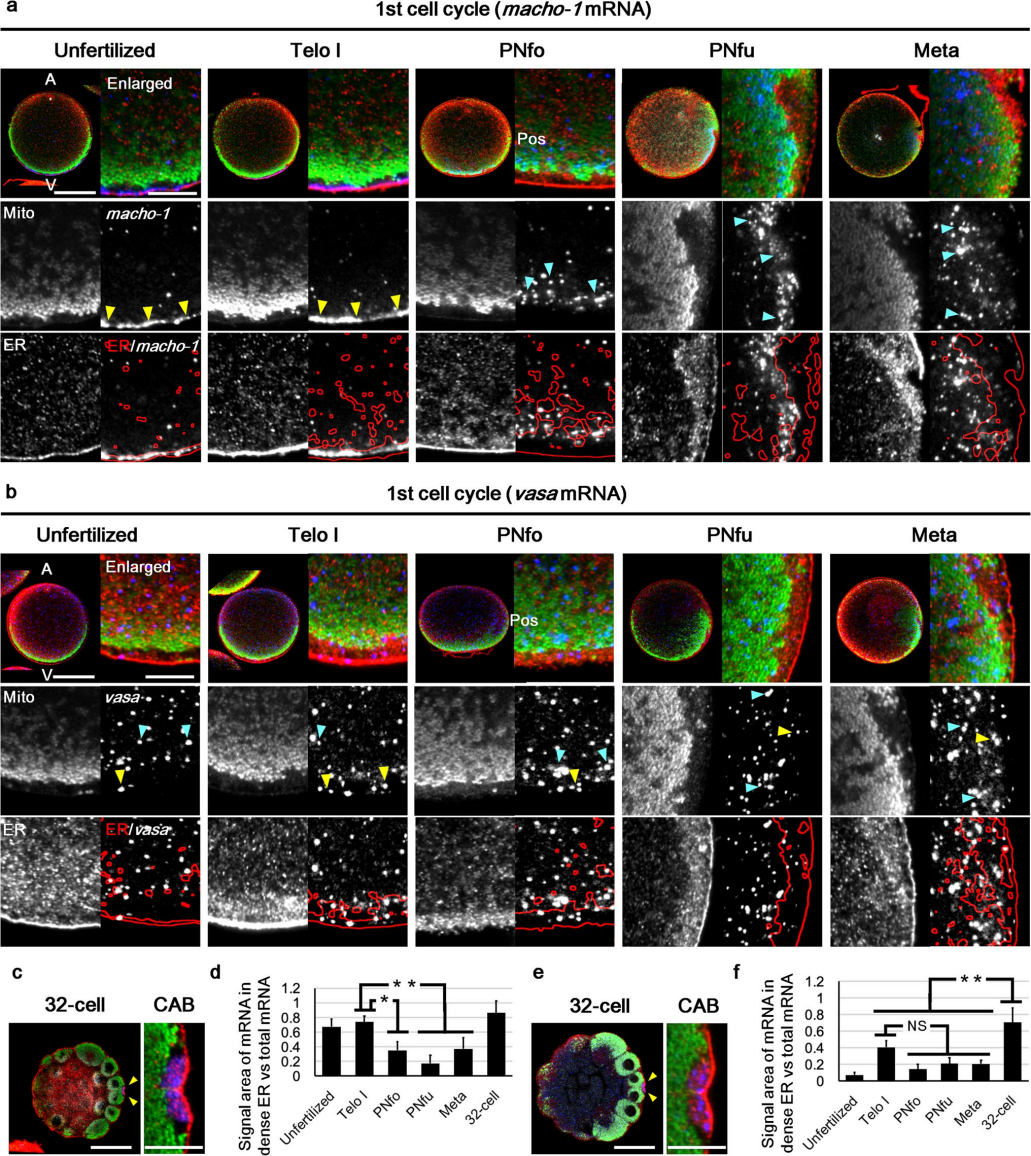
Spatio-temporal pattern of dense ER and postplasmic/PEM RNAs. **a**, **b** Double immunostaining of ER (red) and MRC (green) and in situ hybridisation of maternal mRNAs (blue; *macho-1* **(a)** and *vasa* **(b)**) were performed on the same embryo during the first cell cycle (developmental stages are indicated on the top; unfertilised, Telo I, PNfo, PNfu, and Meta). The optical sections of the mid-plane are shown. Animal pole (A) is up and vegetal pole (V) is down in all photographs. As the antero-posterior axis becomes evident from PNfo stage, posterior pole (Pos) is at the right from this stage onward. Upper tier: merged images of entire egg (no label) and enlarged dense ER region (enlarged). The nucleus (white in the merged images) was counterstained with 4′,6-diamidino-2-phenylindole dihydrochloride. Middle and lower tiers: mitochondria (Mito), ER, and in situ hybridisation (*macho-1* and *vasa*) fluorescence channels of each enlarged image are separately represented (as indicate in the upper-left corner). Outlines of the densely stained ER region (red lines) were superimposed on in situ hybridisation signals (ER/*macho-1* and ER/*vasa*). Most of the *macho-1* signals overlapped with the dense ER region in the unfertilised egg and Telo I stage (yellow arrowheads); however, they were excluded from the dense ER region and extruded into MRC region after the PNfo stage (light blue arrowheads). In contrast, *vasa* signals were detected evenly in the entire cytoplasm and gradually localised to the posterior side; however, they were sparse in the dense ER region. Scale bars: 50 µm (entire egg image) and 10 µm (enlarged image). **c, e** Localisation patterns of maternal mRNAs (blue) at the 32-cell stage were co-stained for ER (red) and mitochondria (green) using our new method. Enlarged images of the CAB (arrowheads) show blotchy staining of ER (red) and mRNA signals (blue) of *macho-1* **(c)** and *vasa* **(e)** within the CAB. Scale bars: 50 µm (left image) and 10 µm (right image). **d, f** Colocalisation between maternal mRNAs and dense ER was quantitatively evaluated by calculating the ratio of the signal area of the *macho-1* **(d)** or *vasa* **(f)** mRNA within the dense ER region to the total area of mRNAs. Statistical significance was calculated by one-way ANOVA followed by the Tukey–Kramer test. Significant differences are represented by symbols (NS no significant difference, *p < 0.05, or **p < 0.01). Error bars represent SD (*n* = 3 with *macho-1*, *n* = 4 with *vasa*)

Next, ER, mitochondria, and postplasmic/PEM RNAs were observed in the same specimen (Fig. 5 and Online Resource 3, Fig. S3). An example of type I postplasmic/PEM RNAs, *macho-1* mRNA, was localised in the egg cortex and condensed at the vegetal pole after the first phase of movement. During this movement, *macho-1* was colocalised with dense ER. After the PNfo stage, *macho-1* exited the vegetal cortex and intruded into the MRC and further into the inner cytoplasm (Fig. 5). Another example of type I postplasmic/PEM RNAs, *pem-1*, showed a translocation movement similar to *macho-1* (Online Resource 3, Fig S3a). In addition, the colocalisation of *macho-1* and cER was re-examined. Isolated cortices were prepared from embryos at 45 mpf (PNm to Prometa) according to the method reported by Sardet et al. (1992, 2011), and quadruple-stained for microtubules, mitochondria, ER, and *macho-1*. We identified the posterior-vegetal cortex by the region, which was rich in microtubule bundles as the remnant of CAMP, and the dislocated MRC next to the posterior-vegetal cortex using mitochondrial staining. Signals of *macho-1* were observed in the MRC region instead of the ER in the posterior-vegetal cortex, regardless of the experimental method (Online Resource 3, Fig. S4). These data support the result that *macho-1* dissociated from dense ER and extruded into the MRC during the second phase of movement.

In contrast, another type II postplasmic/PEM RNA, *vasa* mRNA, which was evenly distributed in the egg cytoplasm, gradually accumulated in the vegetal cortex until the PNfo stage and then to the posterior pole during the second phase of movement. Although a few colocalised signals were observed in the dense ER region, *vasa* did not generally colocalise with the dense ER at the 1-cell stage. Despite the different translocation movements of *macho-1*, *pem-1*, and *vasa* mRNAs during the ooplasmic movement, these signals were similarly observed in the CAB at the 32-cell stage (Fig. 5c, e, and Online Resource, Fig. S3b). Quantitative analysis of the dense ER and mRNA colocalisation clearly indicated these temporal changes (Fig. 5d, f).

### Re-localisation of postplasmic/PEM RNAs in dense ER at the 2-cell stage

**Fig. 6:**
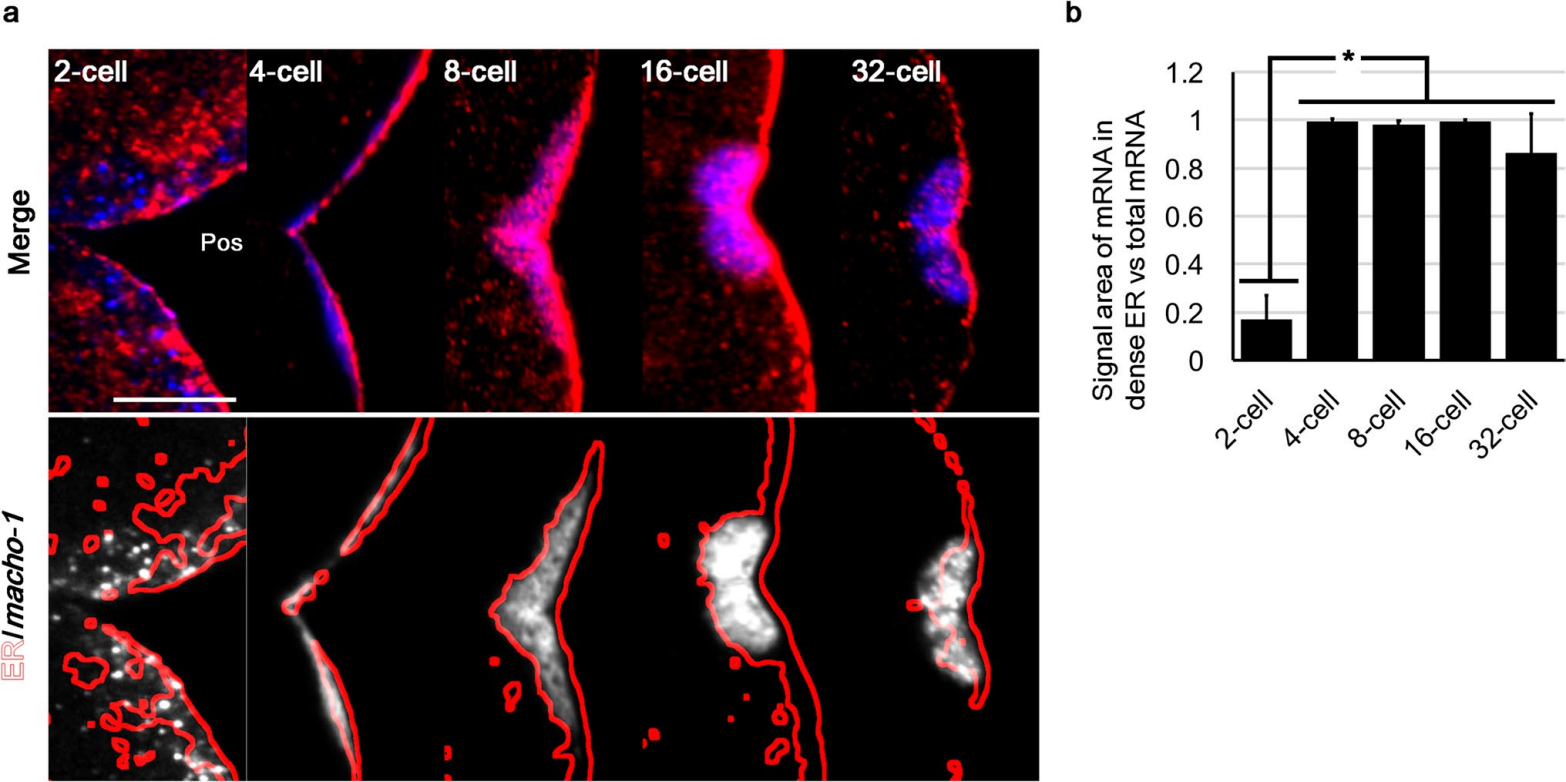
Spatio-temporal pattern of *macho-1* during the cleavage stages. **a** Embryos from 2- to 32-cell stages were stained for ER (red) and *macho-1* (blue) by our new method and counterstained with DAPI. The cell cycle of 2-cell stage was interphase and those of 4-cell stage onward were metaphase. Enlarged images of the CAB-forming regions are shown (merge). Outlines of the densely stained ER region (red lines) were superimposed on *macho-1* signals (white: ER/*macho-1*). Scale bar: 10 µm. **b** Colocalisation between *macho-1* and dense ER was quantitatively evaluated by calculating the ratio of the signal area of the *macho-1* within the dense ER region to the total area of mRNA. Statistical significance was calculated by one-way ANOVA followed by the Tukey–Kramer test. Significant differences are represented by an asterisk (*p* < 0.05). Error bars represent SD (*n* = 5)

We found that postplasmic/PEM RNAs detached from the dense ER before the second movement and re-localised to the dense ER within the CAB before the 32-cell stage. Thus, we stained the ER and postplasmic/PEM mRNAs from the 2- to 32-cell stages (Fig. 6a and Online Resource 3, Fig. S5). At the early 2-cell stage, the dense ER was broadly localised around the posterior cortex, and almost no *macho-1* signal was observed within the dense ER. In contrast, at the 4-cell stage, the dense ER was concentrated in the posterior cortex, and *macho-1* was clearly localised in the dense ER region. At the 8- to 32-cell stages, the dense ER gradually accumulated to the posterior pole and formed the CAB, with *macho-1* showing perfect colocalisation with it (Fig. 6a: 4–32 cells). Quantitative analysis clearly showed that the re-localisation of *macho-1* to the dense ER occurred between the 2- and 4-cell stages (Fig. 6b). When *vasa* and ER were stained from the 2- to 32-cell stages (Online Resource 3, Fig. S5), most of the *vasa* signals were excluded at the early 2-cell stage and localised with dense ER from the 4-cell stage onwards. This translocation movement was similar to that of *macho-1*.

To reveal the timing of the postplasmic/PEM mRNA re-localisation with greater precision, maternal mRNAs and ER were double-stained during the first three rounds of cleavage (Online Resource 3, Fig. S6). Most of the *macho-1* was excluded from dense ER during the mitotic phase of the first cleavage and interphase of the 2-cell stage, while it colocalised with dense ER, situated at the cell cortex of the posterior pole in the mitotic phase of the second cleavage. During the third cleavage, colocalisation between *macho-1* and dense ER was maintained in the CAB-forming region in both the interphase and mitotic phases. This result clearly showed that *macho-1* returned to the dense ER at the 2-cell stage.

## Discussion

The ascidian egg myoplasm has been researched for more than 100 years because of its importance in understanding axis determination and tissue differentiation (Conklin 1905). This long research history has revealed the importance of maternal information, designated as postplasmic/PEM RNAs, and their translational regulation mechanisms (Prodon et al. 2007). Although the ER and ribosomes are organelles essential for translational regulation, the ER in the ascidian egg has been poorly characterized, except in studies using the isolated cortex (Sardet et al. 1992, 2003; Prodon et al. 2005). Thus, we aimed to re-examine the ER of ascidian egg and describe the cortical distribution in detail, especially the relationship between the ER and mRNAs.

In this study, we developed three different immunostaining methods for visualising the entire ER in whole-mount specimens: two of them were double staining methods for ER with mitochondria or microtubules, and the third was for immunostaining the ER with in situ hybridisation. The details of these protocols can be readily shared and are expected to be helpful in developmental research. Using these protocols, we defined the dense ER, which corresponds to the cER but with a greater internally expanded structure, and revealed the separate distribution of the dense ER and MRC, and suggested a possible mechanism for the dense ER translocation. In addition to these methods, we observed the dense ER using FIB-SEM, leading to the description of a cisterna-type structure of the dense ER for the first time.

The ER is a continuous membrane system that forms a network throughout the cytoplasm and performs several cellular functions. It is typically categorised into rough ER (rER) and smooth ER (sER), composed of ribosome-bound ER sheets and ER tubes without ribosomes, respectively (Westrate et al. 2015). These morphological variations are closely linked to their functions. For example, protein synthesis occurs on the rER, and the storage and efflux of calcium ions are preferentially performed by the sER (Lynes and Simmen 2011; Schwarz and Blower 2016). In contrast, the cER, which is tethered to the plasma membrane and exhibits a hybrid shape of a tube and sheet (Westrate et al. 2015), has been reported in a wide variety of animal species (Terasaki and Jaffe 1991; Kline et al. 1999; Estrada et al. 2003), and executes specialised functions, such as lipid synthesis and transportation at the contact sites between the cER and plasma membrane in yeast (Tavassoli et al. 2013; Quon et al. 2018). Mammalian cells also contain cER, which plays an important role in the transportation of membrane-associated proteins (Fox et al. 2013). Moreover, the cER has been observed in the oocytes of a wide range of species, from mouse to sea urchin, and has been reported to contribute to sperm-induced calcium waves (Terasaki and Jaffe 1991; Kline et al. 1999). cER has also been described in ascidian eggs (Sardet et al. 1992) and comprises ribosome-bound tube and sheet structures on the isolated cortex. In our observation using FIB-SEM and confocal microscopy, cisterna-like structures of dense ER were described, and a close relationship between the dense ER and microtubule structure, CAMP, was suggested. As the transition between the tube and sheet structure of the dense ER should be related to the change in ER functions, understanding the molecular mechanism underlying the structural changes and movement of dense ER will help clarify the axis determination mechanism.

Most importantly, our novel staining methods revealed the dynamically changing association between the dense ER and postplasmic/PEM RNAs. *macho-1* and *pem-1* mRNAs (type I postplasmic/PEM mRNAs) detached from the dense ER and shifted their localisation deeper into the internal cytoplasm during the second phase of movement. A close look at previous studies (Sardet et al. 2003; Paix et al. 2011) revealed that the *HrPEM* and *macho-1* mRNAs in the second phase of *Halocynthia* egg and *pem-1* mRNA in 25 and 45 mpf of *Ciona* egg detached from their cortex, although the authors did not mention these results. Moreover, according to the figures presented in Sardet et al. (2003), the meshwork patterns of *HrPEM* and *macho-1* localisation disappeared after the second movement. These results are equivalent to our results and render our data plausible.

According to our results, type I postplasmic/PEM mRNAs detached from the dense ER between the Telo I and PNfo stages (normalised time: 0.2 to 0.6). This period is between the first and second phases of the ooplasmic movement, during which the myoplasm transitions from actin microfilaments to microtubules (Sawada and Schatten, 1989; Chiba et al., 1999). This concurrence suggests the importance of the associations between these cytoskeletal networks for mRNA translocation. Moreover, although *macho-1* detached from the dense ER during the second phase of movement, it moved posteriorly along with the myoplasm. This result suggests that direct binding of the *macho-1* to the ER does not explain the translocation of the former. A detailed analysis of the associations between mRNAs, cytoskeletons, and RNA-binding proteins is required to understand the mRNA translocation mechanism.

*Vasa* did not colocalise with the dense ER during the 1-cell stage, regardless of the weak localisation to the posterior pole after the second phase of movement. Although type I and II postplasmic/PEM mRNAs show similar localisation patterns in the posterior region at the end of the first cell cycle, the localisation mechanism of these mRNAs is generally thought to be different (Sasakura et al. 2000; Paix et al. 2009). In our descriptions, the localisation patterns of these two mRNAs at PNfu and Meta stages were also different; in our study, *macho-1* was almost entirely excluded from the dense ER region, whereas *vasa* was more evenly distributed in the posterior region. This result supported the idea that type I and II postplasmic/PEM mRNAs have discrete translocation mechanisms during the 1-cell stage.

On the other hand, Yamada (2006) reported that 37 postplasmic/PEM mRNAs in *Ciona* egg, including both types I and II, clearly localised to the posterior region, which is assumed to be the dense ER region, at least from the 4-cell stage. We revealed that *macho-1* and *vasa* were re-localised to the dense ER region during the 2-cell stage or up to the 4-cell stage, respectively. Even if the precise timings of their localisation to the CAB were different, postplasmic/PEM mRNAs could associate with ER at a similar time. This similarity raises the possibility that postplasmic/PEM mRNAs share a similar translocation mechanism during the cleavage stage.

These dynamic changes in the dissociation and reassociation between mRNAs and dense ER raise new questions about the movement of postplasmic/PEM mRNAs and their translational regulation. What is the reason for the exclusion of type I postplasmic/PEM mRNAs from the dense ER region during the second phase of movement? Although it is not known whether postplasmic/PEM mRNAs bind to free ribosomes in the MRC region, it is possible that the translation of these mRNAs is active on the dense ER and becomes dormant after their dissociation from the dense ER. The translational products of *macho-1* have been detected using specific antibodies at the 4-cell stage in *Halocynthia* (Kumano et al. 2010). This translational activity of *macho-1* corresponded with its re-localisation to the dense ER. The translation of *pem-1/PEM* mRNA was initiated during the early stage of the first cell cycle in *Ciona* (Paix et al. 2011) and *Halocynthia* (Negishi et al. 2007). Considering the dormancy of unfertilised eggs, these *pem-1/PEM* mRNA translational activities might correspond with its residence in the dense ER during the first phase of movement. Based on these observations, we hypothesised that translational regulation might be achieved by the association and dissociation between mRNA and dense ER. However, *vasa* re-localised to the dense ER at the 4-cell stage in this study, although previous studies have reported that the translation of *vasa* in *Ciona* was detected at the 32-cell stage (Shirae-Kurabayashi et al. 2006). To summarise, translation of each postplasmic/PEM RNA starts at its own time, suggesting various translational regulation mechanisms. Thus, it is important to reveal the precise spatial distribution of each mRNA and dense ER during the initial stage of development to understand the translational regulation mechanism, which is inevitable for embryonic axis formation and morphogenesis.

Although both *macho-1* (transcription factor) and *vasa* (RNA helicase) do not encode secretory or integral membrane proteins (Raz 2000; Nishida and Sawada 2001), the hypothesis that these RNAs are translated on the ER is highly relevant. Hannigan et al. (2020) strongly insisted that essentially all mRNAs, including those encoding cytosolic proteins, are translated to a significant degree on the ER. Moreover, Reid and Nicchitta (2015) reported that RNA-binding proteins on the ER membrane can bind directly to mRNAs in a ribosome-independent manner, suggesting that diverse translational regulations, such as the protection and preservation of maternal mRNAs, may be performed on the ER. In this study, owing to simultaneous imaging using double-, triple-, or quadruple-staining the same specimen, we provided convincing evidence for the complexity of the ooplasmic movement, which includes the translocation and association of organelles and maternal mRNAs. Moreover, we suggested the involvement of the cytoskeleton in the translocation of mRNAs and the plausible translational regulatory role of dense ER during the course of the ooplasmic movement. Understanding the complexity of ooplasmic movement is a promising way to reveal animal developmental mechanisms, including axis formation, cell differentiation, and morphogenesis.

## Conflict of interest

The authors declare no competing interests.

## Supplementary Information

Below is the link to the electronic supplementary material.

Supplementary file1 (AVI 679 KB)

Supplementary file2 (AVI 217 KB)

Supplementary file3 (PDF 4152 KB)

## Data Availability

Our manuscript has data included as electronic supplementary material.

## References

[CR1] Bole DG, Dowin R, Doriaux M, Jamieson JD (1989). Immunocytochemical localization of BiP to the rough endoplasmic reticulum: evidence for protein sorting by selective retention. J Histochem Cytochem.

[CR2] Chenevert J, Pruliere G, Ishii H (2013). Purification of mitochondrial proteins HSP60 and ATP synthase from ascidian eggs: implications for antibody specificity. PLoS ONE.

[CR3] Chiba S, Miki Y, Ashida K (1999). Interactions between cytoskeletal components during myoplasm rearrangement in ascidian eggs. Dev Growth Differ.

[CR4] Conklin EG (1905). The organization and cell lineage of the ascidian egg. J Acad Nat Sci Phila.

[CR5] Estrada P, Kim J, Coleman J (2003). Myo4p and She3p are required for cortical ER inheritance in Saccharomyces cerevisiae. J Cell Biol.

[CR6] Fox PD, Haberkorn CJ, Weigel AV (2013). Plasma membrane domains enriched in cortical endoplasmic reticulum function as membrane protein trafficking hubs. Mol Biol Cell.

[CR7] Goto T, Kanda K, Nishikata T (2019). Non-centrosomal microtubule structures regulated by egg activation signaling contribute to cytoplasmic and cortical reorganization in the ascidian egg. Dev Biol.

[CR8] Hannigan MM, Hoffman AM, Thompson JW (2020). Quantitative proteomics links the LRRC59 interactome to mRNA translation on the ER membrane. Mol Cell Proteomics.

[CR9] Hayashi T, Lewis A, Hayashi E (2011). Antigen retrieval to improve the immunocytochemistry detection of sigma-1 receptors and ER chaperones. Histochem Cell Biol.

[CR10] Ibrahim IM, Abdelmalek DH, Elfiky AA (2019). GRP78: a cell’s response to stress. Life Sci.

[CR11] Ishii H, Kunihiro S, Tanaka M (2012). Cytosolic subunits of ATP synthase are localized to the cortical endoplasmic reticulum-rich domain of the ascidian egg myoplasm. Dev Growth Differ.

[CR12] Ishii H, Shirai T, Makino C, Nishikata T (2014). Mitochondrial inhibitor sodium azide inhibits the reorganization of mitochondria-rich cytoplasm and the establishment of the anteroposterior axis in ascidian embryo. Dev Growth Differ.

[CR13] Ishii H, Goto T, Nishikata T (2017). Microtubule array observed in the posterior-vegetal cortex during cytoplasmic and cortical reorganization of the ascidian egg. Dev Growth Differ.

[CR14] Kline D, Mehlmann L, Fox C, Terasaki M (1999). The cortical endoplasmic reticulum (ER) of the mouse egg: localization of ER clusters in relation to the generation of repetitive calcium waves. Dev Biol.

[CR15] Kumano G, Kawai N, Nishida H (2010). Macho-1 regulates unequal cell divisions independently of its function as a muscle determinant. Dev Biol.

[CR16] Lyczak R, Gomes JE, Bowerman B (2002). Heads or tails: cell polarity and axis formation in the early Caenorhabditis elegans embryo. Dev Cell.

[CR17] Lynes EM, Simmen T (2011). Urban planning of the endoplasmic reticulum (ER): how diverse mechanisms segregate the many functions of the ER. Biochim Biophys Acta.

[CR18] Miller JR, Rowning BA, Larabell CA (1999). Establishment of the dorsal-ventral axis in Xenopus embryos coincides with the dorsal enrichment of dishevelled that is dependent on cortical rotation. J Cell Biol.

[CR19] Nance J, Zallen JA (2011). Elaborating polarity: PAR proteins and the cytoskeleton. Development.

[CR20] Negishi T, Takada T, Kawai N, Nishida H (2007). Localized PEM mRNA and protein are involved in cleavage-plane orientation and unequal cell divisions in ascidians. Curr Biol.

[CR21] Nishida H (1994). Localization of determinants for formation of the anterior–posterior axis in eggs of the ascidian Halocynthia roretzi. Development.

[CR22] Nishida H (2005). Specification of embryonic axis and mosaic development in ascidians. Dev Dyn.

[CR23] Nishida H, Sawada K (2001). macho-1 encodes a localized mRNA in ascidian eggs that specifies muscle fate during embryogenesis. Nature.

[CR24] Nishikata T, Hibino T, Nishida H (1999). The centrosome-attracting body, microtubule system, and posterior egg cytoplasm are involved in positioning of cleavage planes in the ascidian embryo. Dev Biol.

[CR25] Paix A, Yamada L, Dru P (2009). Cortical anchorages and cell type segregations of maternal postplasmic/PEM RNAs in ascidians. Dev Biol.

[CR26] Paix A, Le Nguyen PN, Sardet C (2011). Bi-polarized translation of ascidian maternal mRNA determinant pem-1 associated with regulators of the translation machinery on cortical endoplasmic reticulum (cER). Dev Biol.

[CR27] Prodon F, Dru P, Roegiers F, Sardet C (2005). Polarity of the ascidian egg cortex and relocalization of cER and mRNAs in the early embryo. J Cell Sci.

[CR28] Prodon F, Yamada L, Shirae-Kurabayashi M (2007). Postplasmic/PEM RNAs: a class of localized maternal mRNAs with multiple roles in cell polarity and development in ascidian embryos. Dev Dyn.

[CR29] Prodon F, Hanawa K, Nishida H (2009). Actin microfilaments guide the polarized transport of nuclear pore complexes and the cytoplasmic dispersal of Vasa mRNA during GVBD in the ascidian Halocynthia roretzi. Dev Biol.

[CR30] Quon E, Sere YY, Chauhan N (2018). Endoplasmic reticulum-plasma membrane contact sites integrate sterol and phospholipid regulation. PLOS Biol.

[CR31] Raz E (2000) The function and regulation of vasa-like genes in germ-cell development. Genome Biol 1:REVIEWS1017. 10.1186/gb-2000-1-3-reviews1017 10.1186/gb-2000-1-3-reviews1017 PMC138859 11178242

[CR32] Reid DW, Nicchitta CV (2015). Diversity and selectivity in mRNA translation on the endoplasmic reticulum. Nat Rev Mol Cell Biol.

[CR33] Roegiers F, Djediat C, Dumollard R (1999). Phases of cytoplasmic and cortical reorganizations of the ascidian zygote between fertilization and first division. Development.

[CR34] Sardet C, Speksnijder J, Terasaki M, Chang P (1992). Polarity of the ascidian egg cortex before fertilization. Development.

[CR35] Sardet C, Nishida H, Prodon F, Sawada K (2003). Maternal mRNAs of PEM and macho 1, the ascidian muscle determinant, associate and move with a rough endoplasmic reticulum network in the egg cortex. Development.

[CR36] Sardet C, Paix A, Prodon F (2007). From oocyte to 16-cell stage: cytoplasmic and cortical reorganizations that pattern the ascidian embryo. Dev Dyn.

[CR37] Sardet C, McDougall A, Yasuo H (2011). Embryological methods in ascidians: the Villefranche-sur-Mer protocols. Methods Mol Biol.

[CR38] Sasakura Y, Ogasawara M, Makabe KW (2000). Two pathways of maternal RNA localization at the posterior-vegetal cytoplasm in early ascidian embryos. Dev Biol.

[CR39] Satou Y, Satoh N (2005). Cataloging transcription factor and major signaling molecule genes for functional genomic studies in Ciona intestinalis. Dev Genes Evol.

[CR40] Sawada T, Schatten G (1989). Effects of cytoskeletal inhibitors on ooplasmic segregation and microtubule organization during fertilization and early development in the ascidian Molgula occidentalis. Dev Biol.

[CR41] Schindelin J, Arganda-Carreras I, Frise E (2012). Fiji: an open-source platform for biological-image analysis. Nat Methods.

[CR42] Schwarz DS, Blower MD (2016). The endoplasmic reticulum: structure, function and response to cellular signaling. Cell Mol Life Sci.

[CR43] Shirae-Kurabayashi M, Nishikata T, Takamura K (2006). Dynamic redistribution of vasa homolog and exclusion of somatic cell determinants during germ cell specification in Ciona intestinalis. Development.

[CR44] Speksnijder JE, Terasaki M, Hage WJ (1993). Polarity and reorganization of the endoplasmic reticulum during fertilization and ooplasmic segregation in the ascidian egg. J Cell Biol.

[CR45] Tao Q, Yokota C, Puck H (2005). Maternal wnt11 activates the canonical Wnt signaling pathway required for axis formation in Xenopus embryos. Cell.

[CR46] Tavassoli S, Chao JT, Young BP (2013). Plasma membrane—endoplasmic reticulum contact sites regulate phosphatidylcholine synthesis. EMBO Rep.

[CR47] Terasaki M, Jaffe LA (1991). Organization of the sea urchin egg endoplasmic reticulum and its reorganization at fertilization. J Cell Biol.

[CR48] Terasaki M, Reese TS (1992). Characterization of endoplasmic reticulum by co-localization of BiP and dicarbocyanine dyes. J Cell Sci.

[CR49] Westrate LM, Lee JE, Prinz WA, Voeltz GK (2015). Form follows function: the importance of endoplasmic reticulum shape. Annu Rev Biochem.

[CR50] Yamada L (2006). Embryonic expression profiles and conserved localization mechanisms of pem/postplasmic mRNAs of two species of ascidian, Ciona intestinalis and Ciona savignyi. Dev Biol.

