# Supplementary material for: Dynamic changes in the association between maternal mRNAs and endoplasmic reticulum during ascidian early embryogenesis
Source: Dev Genes Evol. 2021 Dec 18;232(1):1–14. doi: 10.1007/s00427-021-00683-y (PMC8918112; doi:10.1007/s00427-021-00683-y)
Supplement: Supplementary file 3 — Supplementary file3 (PDF 4152 KB) [file 427_2021_683_MOESM3_ESM.pdf]

Development Genes and Evolution

Online Resource 3
Electronic Supplementary Information for

Dynamic changes in the association between maternal mRNAs and endoplasmic reticulum during ascidian early embryogenesis

Toshiyuki Goto[1, 3], Shuhei Torii[1], Aoi Kondo[1], Junji Kawakami[1], Haruka Yagi[2], Masato Suekane[2], Yosky Kataoka[3, 4], Takahito Nishikata[1*]

[1]Frontiers of Innovative Research in Science and Technology (FIRST), Konan University, Kobe, Hyogo 650-0047, Japan

[2]Japan Testing Laboratories, Inc, Kobe, Hyogo 654-0161, Japan

[3]Laboratory for Cellular Function Imaging, RIKEN Center for Biosystems Dynamics Research, Kobe, Hyogo 650-0047, Japan

[4]Multi-Modal Microstructure Analysis Unit, RIKEN-JEOL Collaboration Center, Kobe, Hyogo 650-0047, Japan

* Corresponding author: T. Nishikata

E-mail: nisikata@konan-u.ac.jp

Address: 7-1-20 Minatojima-minamimachi, Chuo-ku, Kobe 605-0047, Japan.

Tel: +81-78-303-1349; Fax: +81-78-303-1495

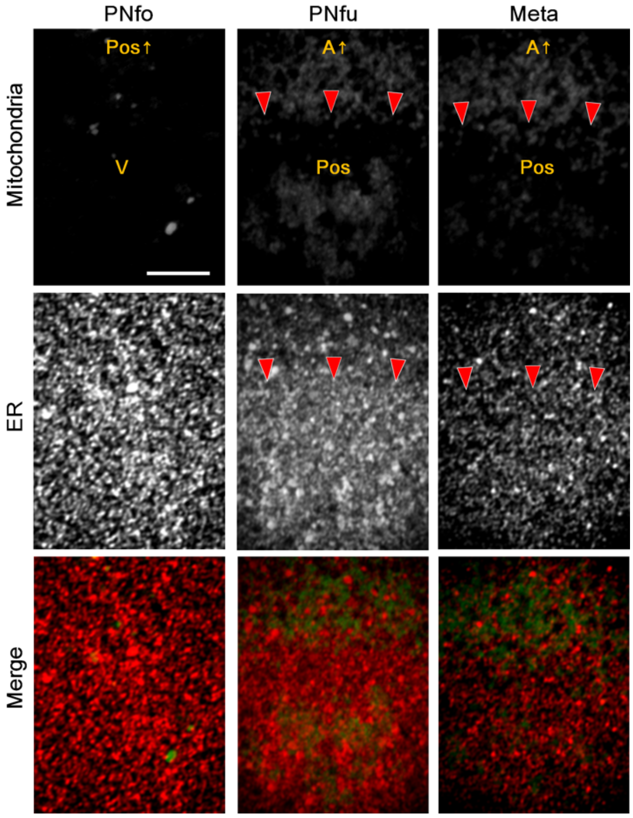

Fig. S1 Vegetal or posterior views of Fig. 2c. A 3D model of approximately 4 μm thickness was rendered from optical sections of high-magnification images of dense ER regions at PNfo, PNfu, and Meta (as indicated on the top). Mitochondria, ER, and merged fluorescence channels are separately shown (as indicated on the left). PNfo: Vegetal view of vegetal pole region is indicated with posterior side (Pos↑) up. Dense ER is visible; however, MRC cannot be observed in this 3D model. PNfu and Meta: Posterior views of posterior pole region (Pos) are indicated with animal pole side (A↑) up. The animal pole side borders of dense ER (arrowheads), which was peeled off from the egg cortex and started to intrude into cytoplasm, are obvious. Bright dots in the ER channel can be seen just beneath the plasma membrane. Scale bar: 5 μm.

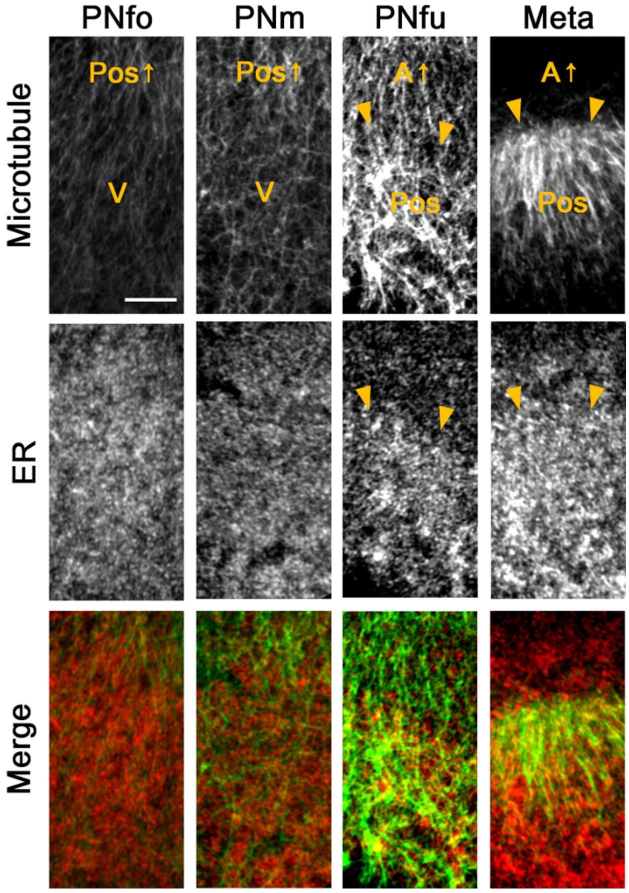

Fig. S2 Vegetal or posterior views of Fig. 3c. The 3D model of approximately 18 μm thickness was rendered from optical sections of high-magnification images of dense ER regions at PNfo, PNm, PNfu, and Meta (as indicated on the top). Microtubules, ER, and merged fluorescence channels are separately shown (as indicated on the left). PNfo and PNm: Vegetal view of vegetal pole region are indicated with posterior side (Pos↑) up. At the vegetal pole, dense ER is obvious in PNfo and becomes faint in PNm, while cortical array of microtubule in posterior-vegetal region (CAMP) starts to form at the PNm stage. PNfu and Meta: Posterior views of posterior pole region (Pos) are indicated with animal pole side (A↑) up. The animal pole side borders of the dense ER (arrowheads), peeled off from the egg cortex and starting to intrude into the cytoplasm, are obvious. Bright dots in the ER channel are visible just beneath the plasma membrane. The parallel array of microtubules in CAMP is obvious and shortened at the Meta stage. Scale bar: 10 μm.

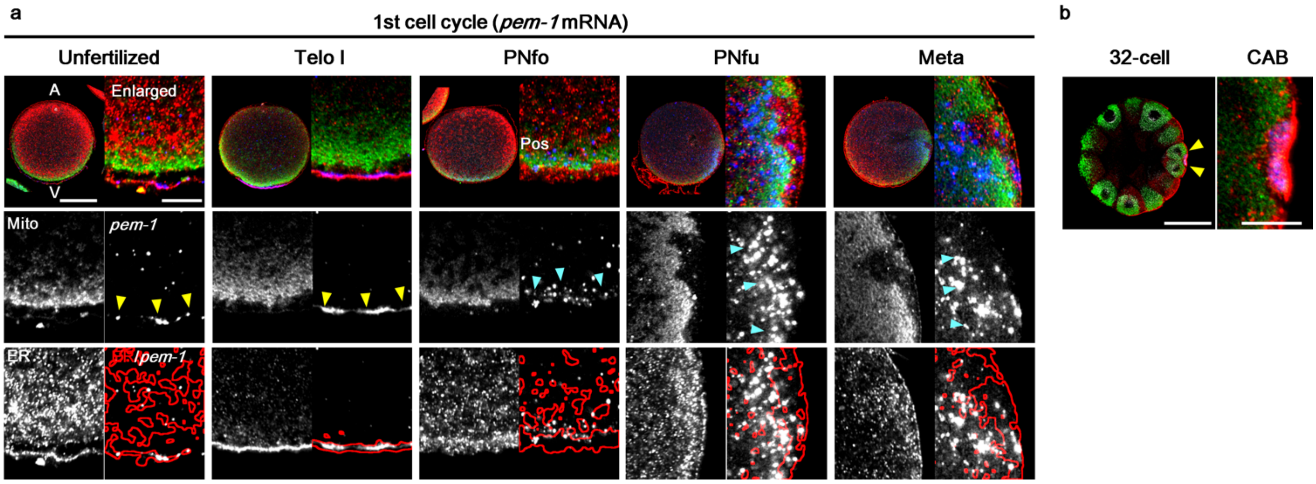

Fig. S3 Spatio-temporal pattern of dense ER and *pem-1* mRNA. **(a)** Double-immunostaining of ER (red) and MRC (green) and *in situ* hybridisation of another example of type I postplasmic/PEM mRNAs (blue; *pem-1*), were performed on the same embryo during the first cell cycle (developmental stages are indicated on the top; unfertilised, Telo I, PNfo, PNfu, and Meta). The optical sections of the mid-plane are shown. Animal pole (A) is up and vegetal pole (V) is down in all photographs. As the antero-posterior axis becomes evident from the PNfo stage, posterior pole (Pos) is at the right from this stage onward. Upper tier: Merged images of entire egg (no label) and enlarged dense ER region (Enlarged) are shown. The nucleus (white in the merged images) was counterstained with 4',6-Diamidino-2-phenylindole dihydrochloride. Middle and lower tiers: Mitochondria (Mito), ER, and *in situ* hybridisation (*pem-1*) fluorescence channels of each enlarged images are separately represented (as indicated in the upper-left corner). Outlines of the densely stained ER region (red lines) were superimposed on *in situ* hybridisation signals (ER/*pem-1*). Most of the *pem-1* mRNA signals overlapped with the dense ER region in the unfertilised egg and Telo I stage (yellow arrowheads); however, they were excluded from the dense ER region and extruded into the MRC region after the PNfo stage (light blue arrowheads). Scale bar: 50 μm (entire egg image) and 10 μm (enlarged image). **(b)** Localisation patterns of *pem-1* mRNAs (blue) at the 32-cell stage were co-stained for ER (red) and mitochondria (green) using our new method. Enlarged images of the CAB (arrowheads) show blotchy staining of ER (red) and *pem-1* mRNA signals (blue) within the CAB. Scale bars: 50 μm (left image) and 10 μm (right image).

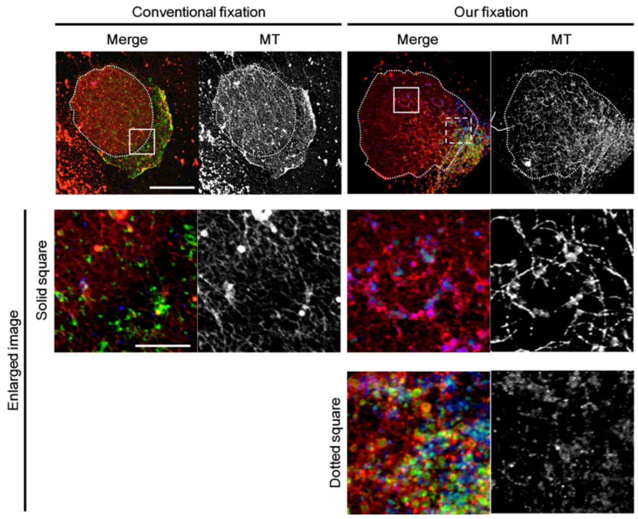

Fig. S4 Spatial pattern of ER and *macho-1* mRNA in the isolated cortices. Cortices isolated from embryos at 45 mpf were quadruple-stained for ER (red), *macho-1* (green), mitochondria (blue), and microtubule (MT; white) by the conventional or our new experimental method (as indicated on the top). The 3D models of approximately 4 μm thickness are rendered from optical sections. Merged images (Merge) and microtubule fluorescent channel (MT) are shown. These cortices were assumed to be sheared by the water flow from upper-left corner; thus, the dislocated MRCs were located at the lower-right side of each isolated cortex (dotted line). Enlarged images of solid and dashed rectangles area are shown (Enlarged image; as indicated on the left side). Upper-left and lower-right halves of both solid rectangle areas represent posterior-vegetal cortex and MRC region, respectively. The dotted rectangle represents the rim of ER-rich region, which is assumed to correspond with the dense ER. With the conventional method, posterior-vegetal cortex was rich in ER, whereas the signals of *macho-1* mRNA were more evident in the MRC region. With our method, the signals of *macho-1* mRNA predominantly reside in the MRC region and are rarely observed on the posterior-vegetal cortex. Scale bars: 50 μm (upper tier) and 10 μm (enlarged images).

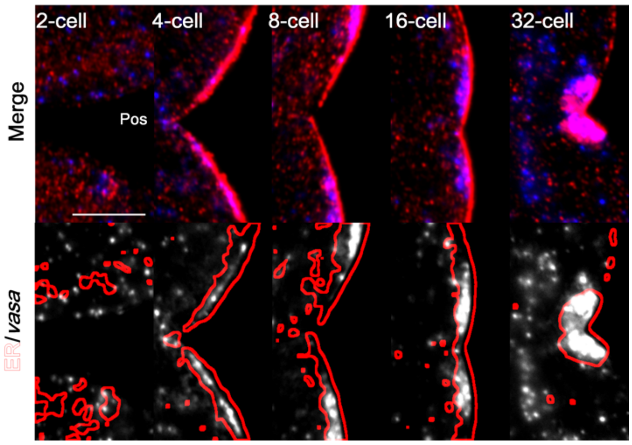

Fig. S5 Spatio-temporal pattern of *vasa* mRNA during the cleavage stages. Embryos from 2- to 32-cell stages were stained for ER (red) and *vasa* (blue) with our new method and counterstained with DAPI. Cell cycle of 2- to 16-cell stages were interphase and those of 32-cell stage was metaphase. Enlarged images of the CAB-forming regions are shown (Merge). Outlines of the densely stained ER region (red lines) were superimposed on the *vasa* signals (white: ER/*vasa*). Most of the *vasa* mRNA (type II postplamic/PEM mRNA) signals were colocalised with dense ER from the 4-cell stage, similar to the type I postplasmic/PEM RNA. Scale bar: 10 μm.

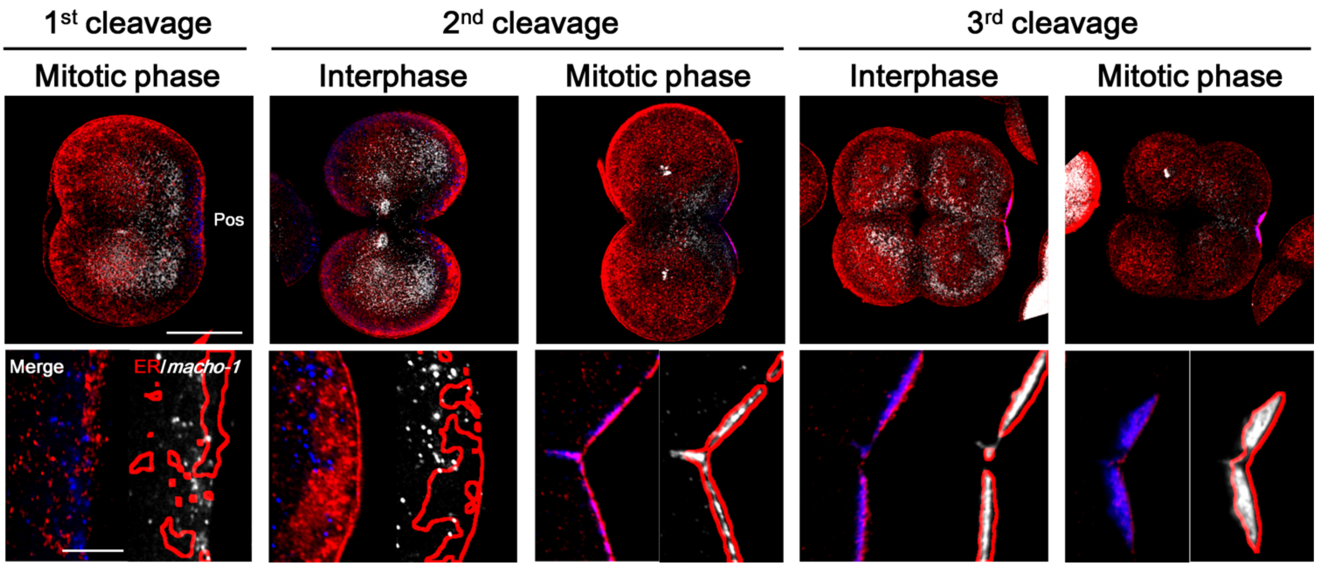

Fig. S6 Spatio-temporal pattern of macho-1 mRNA from the first to third cleavage stage. To reveal when the *macho-1* mRNA was relocalised with dese ER, embryos in the first mitotic phase (first cleavage), interphase and mitotic phase of the 2-cell stage (second cleavage), and interphase and mitotic phase of the 4-cell stage (third cleavage) were stained for ER (red) and *macho-1* (blue), and counter stained with DAPI (white). Upper tier: Merged images of equatorial plane are shown. Posterior pole (Pos) is at the right. Lower tier: Enlarged images of presumptive CAB-forming regions are represented in two ways: simply enlarged image (Merge) and image with outlines of the densely stained ER region (red lines) on *macho-1* signals (white: ER/*macho-1*). Most of the *macho-1* mRNA signals were excluded from dense ER region until interphase of 2-cell stage, whereas at the metaphase of second mitosis (2- to 4-cell stage), most of the mRNA signals were colocalised with the dense ER at the posterior pole. Scale bars: 50 μm (upper tire) and 10 μm (lower tier).
